# Molecular Imaging in Breast Cancer

**DOI:** 10.1155/2012/426260

**Published:** 2012-12-29

**Authors:** Alvaro Ruibal, José María Benlloch, Renato Valdés Olmos, Bengt Langstrom

**Affiliations:** ^1^IDIS, Complejo Hospitalario Universitario, Universidade de Santiago de Compostela, 15706 Santiago de Compostela, Spain; ^2^Instituto de Instrumentación para Imagen Molecular, Universidad Politécnica de Valencia (UPV) and Consejo Superior de Investigaciones Científicas (CSIC), 46022 València, Spain; ^3^Nuclear Medicine Department, The Netherlands Cancer Institute-Antoni van Leeuwenhoek Hospital, 1066 CX Amsterdam, The Netherlands; ^4^Physical Organic Chemistry, Department of Chemistry (BMC), Uppsala University, 75123 Uppsala, Sweden

## 1. Breast Cancer: Challenges in Diagnosis and Characterization Using Molecular Imaging Techniques

At the present time, *breast cancer (BC)* is one of the most frequently diagnosed cancers and the leading cause of cancer death in females worldwide. Breast cancer is a heterogeneous disease including different groups of illnesses in relation to presentation, biology, clinical behavior, and response to therapy. Thus, nowadays, knowledge of their biology is imposed in daily practice. Likewise, we know that tumor biology can also affect significantly the efficacy of the different modern techniques of molecular imaging.

While many breast cancers are not associated with known risk factors, others do, highlighting with a relative risk [(RR) > 4] the following: *females; age:* >65 *versus* <65 years; *gene mutations* (BRCA1 and BRCA2);* two or more first degree relatives with BC in early ages; personal history of BC*; *high breast tissue density and biopsy-confirmed atypical hyperplasia (Breast Cancer Facts & Figures  2009-2010). *In relation with secondary prevention, we know that aspirin and other nonsteroidal anti-inflammatory drugs (NSAIDs) inhibit production of prostaglandins and cyclooxygenase 2, and they are potential agents for chemoprevention of breast cancer. Ameta-analysis indicates that regular use of aspirin may be associated with reduced risk of breast cancer (OR: 0.86) [1].

With advances in the sensitivity of mammographic screening and the broader population of women screened via national programs in different countries, the diagnosis of breast tumors has changed considerably. As an example, more than 50% of all invasive breast cancers in the UK are screen-detected and 52.3% of all invasive breast cancers measured 15 mm or less in diameter and were deemed clinically nonpalpable [2]. Likewise, about 20–30% of the new breast cancers are in situ carcinomas, and 60–80% of all invasive breast cancers have no axillary lymph node involvement (N), so we need to have new prognostic factors other than classics ones (size, N, and distant metastases (M)). *Screening-detected breast cancer* is associated with older age, smaller tumor size, more hormone receptor positiveness, less lymph node involvement, earlier stage, and reduced mortality compared with symptomatic breast cancer. According to the molecular subtype, luminal A is the most common (63.6%) and acts as an independent prognostic factor itself. Another aspect of practical interest is interval cancers, those detected between two screening scans. *Interval cancer* represents between 12% and 28% of all cancers diagnosed in a screening program, especially in women below 50 years, and have a higher percentage of nonductal histology. Likewise, they often overexpressed epidermal growth factor receptor (EGFR) and were more frequently estrogen receptor negative and triple negative, with a poorer prognosis. This observationprovides new data indicating that EGFR may be important in the etiology of interval cancer.

The standardimaging techniques for the diagnosis of this malignant tumor includes compression and magnification mammography (digital or not), breast ultrasounds (USs), and MRI. Although PET/CT is not included in initial explorations of breast cancer, it is known to be useful in detecting distant metastasis (7-8% of patients who are not suspected) and to improve the prediction of neoadjuvant therapy response. Recently, tomosynthesis and PEM (positron emission mammography)have been included as diagnostictoolsin a reduced number of medical centers.

The problem of imaging techniques focused on the important number of false positive results obtained with mammography, so reduction of false positive riskand associated factors is, nowadays, the major goal of breast cancer screening. Tomosynthesis has improved the diagnostic efficacy, and MRI can play an important role in preoperative planning if used in selected patients with high risk of multifocal/multicentric lesions. Also it is very useful in young women or with dense breast and in BRCA1/2 mutation carriers. However, the histological confirmation of all suspicious findings detected by MRI is compulsory prior to definitive surgery [3].

## 2. Which Problems We Find?

In daily practice, we find the following situations that require an effort from the image scans to be solved. 

### 2.1. Mammograms Doses of Radiation

This technique requires very small doses of radiation (2–4 mGy; 200–400 mRad). The risk is slight, but repeated X-rays have the potential to cause cancer, but this is very rare. However, we know the mechanisms of DNA repair after ionizing radiation and many of the genes involved are altered in certain subpopulations that we need to keep in mind. These patients show a greater sensitivity to ionizing radiation and the continued use of this imaging technique can be harmful. 

Special interest have patients with mutations in BRCA1and/or BRCA2 genes and ataxia telangiectasia (ATM) gene, as well as certain genes of Fanconi anemia and those related to the normal functioning of the cell cycle. Another aspect of great value is the absorbed dose by the mammary glands of girls and young women during radiological examinations of other sites of their bodies. As an example, the chest CT and abdominal CT scan radiate between 3 and 30 times the dose of a mammogram. We need to take into account the future consequences of these “unnecessary” radiations.

### 2.2. Occult Breast Cancer Presenting with an Axillary Metastasis

Occult breast cancer incidence is decreasing, because more primary breast cancers can be detected with the introduction of more advanced techniques. Breast MRI can identify the primary tumor in approximately two thirds of this population, but, due to the low specificity, lesions need to be histologically confirmed. Other recent imaging techniques can be useful also. The immunohistochemical study of the lymph node using certain breast cyst fluid proteins (alpha-2-glycoprotein 1, zinc-binding, apolipoprotein D, GCDFP15, etc.) may be of great diagnostic value. 

### 2.3. Nonpalpable Breast Cancer

This is a situation of great practical interest, and its location is a routine necessity. There are many techniques for identification, and we know that US-guided BCS (breast conserving surgery) could be more accurate than wire-guide localization (WL) and ROLL-guided surgery because it optimized the surgeon's ability to obtain adequate margins. However, other authors consider that the radioguided localization surgery determines lower positive margins rates and fewer reoperations.

### 2.4. Ductal Carcinoma In Situ in Younger Women

Ductal carcinomas in situ (DCIS) are a heterogeneous entity and early and accurate diagnosis is a necessity, since they are often tumors in women under 40 years. Likewise, high recurrence rates(mainly invasive: 62%; in situ: 38%) after breast conservative treatment have been observed in young women with this tumor.

### 2.5. Sentinel Node (SLNB)

At present, their detection and analysis is a mandatory practice in the breast cancer surgery. The use of radioguided methods for lymphatic mapping and SLNB localization has represented a breakthrough. There are several nomograms for predicting the nonsentinel lymph node metastasesafter a positive sentinel lymph node biopsy and also we know that lymphoedema occurs in 21% of patients who undergo axillary lymph node dissection (ALND) versus 7% of patients who undergo SLNB. Optical scans seem to be reliable and effective to detect this lymph node when located in level one of the axilla. The clinical significance of micrometastases continues to be a subject of controversy, even though now some groups consider that they do not influence tumor outcome.

### 2.6. Evaluation of Axillary Lymph Node Metastases

The accurate diagnosis of axillary nodal involvement using imaging methods is of great practical usefulness. SLNB remains the standard of care to stage the axilla. MRI could be the most cost-effective strategy to replace SLNB. However, further large studies are required to obtain more accurate data on the sensitivity and specificity of this technique [4]. Gadolinium-enhanced MRI has a sensitivity of 88% and a specificity of 100%, and ultrasmall superparamagnetic iron oxide-(USPIO-) enhanced MRI has 98% and 96% respectively. However, it does not appear that currently can replace SLNB. Axillary ultrasound fine needle aspiration has a sensitivity of 63.4% and a specificity of 100%, while intraoperative frozen section analysis of the sentinel node has a sensitivity of 76.5% and a specificity of 100%. Sensitivity of both procedures together is 91.4%. In patients with stage II and III breast cancer, high-resolution FDG PET/CT has reached a sensitivity of 82% and a specificity of 92% in detecting axillary metastases, and due to its high predictive value (98%), if positive, SLNB may be omitted [5].

### 2.7. Locoregional Breast Cancer Recurrence (LRR)

Luminal tumors exhibit the lowest rates of locoregional recurrence while patients with triple negative and HER2 overexpression have an increased risk of developing LRR following surgery and other associated treatments. In this clinical situation, FDG-PET/CT can be a good choice, with a sensitivity, specificity, accuracy, and positive and negative predictive values of 97%, 92%, 95%, and 94% and 96%, respectively. Also,this technique can change the clinical management in almost half of the patients. The use of new PET tracers opens a new point of view giving us more physiopathological information. Also the “classic” imaging techniques (mammography US), MRI, and PEM can play an important role is this clinical situation.

### 2.8. Identification of Residual Breast Tumor Localization after Neoadjuvant Chemotherapy

It is an increasing clinical situation due to the enhancing of conservative surgery. Besides the current imaging techniques, recently has been introduced in clinical practice the use of 125I seed and its injection into the tumor area before chemotherapy with very good results, due to the half time of 125I (60 days).

## 3. Challenges

*The first challenge* posed is to diagnose and establish the stage of breast cancer in its earliest stages and as safely as possible. Also the study of premalignant lesions will be crucial in the near future. We must focus our efforts in these different areas to make no delay diagnosis. In relation with this, in Sweden, the delay in diagnosis occurs in about 1/1000 new patients and the delay was considered to have an impact on the magnitude of therapeutic measures in almost 25% of the women. Economic compensation for the patient's injuries was given in 90% of the cases [6]. *The second challenge* is the detection of a recurrence prior to the development of symptoms, which can improve overall survival. *The third challenge* will be to study how the biology of the tumor is capable of influence imaging techniques, especially the molecular imagines, and how we define those targets that allow us the biological characterization of an injury through an image. The use of modern laboratory techniques (stem cells, proteomics, epigenetic, etc.) will be mandatory. The use of advance imaging technology is a necessity and a reality, but, as Panageas et al. write [7], a question arises in some situations: *“It is not clear whether if early detection of distant recurrence leads to a survival benefit improvement or whether it simply increases the amount of time women spend living with the knowledge that their disease has recurred.”*

Finally, we thank the different authors for their hard work and excellent papers that allowed the elaboration of this special issue. Our main goal was to show different aspects of breast cancer related to biology and molecular imaging as well as their clinical usefulness.

*Alvaro Ruibal* *Alvaro Ruibal* *José María Benlloch* *José María Benlloch* *Renato Valdés Olmos* *Renato Valdés Olmos* *Bengt Langstrom* *Bengt Langstrom*
